# Voltage-Dependent Anion Channel 1 As an Emerging Drug Target for Novel Anti-Cancer Therapeutics

**DOI:** 10.3389/fonc.2017.00154

**Published:** 2017-07-31

**Authors:** Varda Shoshan-Barmatz, Yakov Krelin, Anna Shteinfer-Kuzmine, Tasleem Arif

**Affiliations:** ^1^Department of Life Sciences, National Institute for Biotechnology in the Negev, Ben-Gurion University of the Negev, Beer-Sheva, Israel

**Keywords:** apoptosis, cancer, metabolism, mitochondria, voltage-dependent anion channel 1

## Abstract

Cancer cells share several properties, high proliferation potential, reprogramed metabolism, and resistance to apoptotic cues. Acquiring these hallmarks involves changes in key oncogenes and non-oncogenes essential for cancer cell survival and prosperity, and is accompanied by the increased energy requirements of proliferating cells. Mitochondria occupy a central position in cell life and death with mitochondrial bioenergetics, biosynthesis, and signaling are critical for tumorigenesis. Voltage-dependent anion channel 1 (VDAC1) is situated in the outer mitochondrial membrane (OMM) and serving as a mitochondrial gatekeeper. VDAC1 allowing the transfer of metabolites, fatty acid ions, Ca^2+^, reactive oxygen species, and cholesterol across the OMM and is a key player in mitochondrial-mediate apoptosis. Moreover, VDAC1 serves as a hub protein, interacting with diverse sets of proteins from the cytosol, endoplasmic reticulum, and mitochondria that together regulate metabolic and signaling pathways. The observation that VDAC1 is over-expressed in many cancers suggests that the protein may play a pivotal role in cancer cell survival. However, VDAC1 is also important in mitochondria-mediated apoptosis, mediating release of apoptotic proteins and interacting with anti-apoptotic proteins, such as B-cell lymphoma 2 (Bcl-2), Bcl-xL, and hexokinase (HK), which are also highly expressed in many cancers. Strategically located in a “bottleneck” position, controlling metabolic homeostasis and apoptosis, VDAC1 thus represents an emerging target for anti-cancer drugs. This review presents an overview on the multi-functional mitochondrial protein VDAC1 performing several functions and interacting with distinct sets of partners to regulate both cell life and death, and highlights the importance of the protein for cancer cell survival. We address recent results related to the mechanisms of VDAC1-mediated apoptosis and the potential of associated proteins to modulate of VDAC1 activity, with the aim of developing VDAC1-based approaches. The first strategy involves modification of cell metabolism using VDAC1-specific small interfering RNA leading to inhibition of cancer cell and tumor growth and reversed oncogenic properties. The second strategy involves activation of cancer cell death using VDAC1-based peptides that prevent cell death induction by anti-apoptotic proteins. Finally, we discuss the potential therapeutic benefits of treatments and drugs leading to enhanced VDAC1 expression or targeting VDAC1 to induce apoptosis.

## Voltage-Dependent Anion Channel (VDAC) Isoforms, Structure, and Channel Activity

### VDAC Isoforms and Cellular Localization

In mammals, three VDAC isoforms have been identified, VDAC1, VDAC2, and VDAC3, sharing ~70% identity as well as several structural and functional properties (1, 2), although they are significantly different with relation to functionality (1, 3, 4). The three isoforms are expressed in most tissue types, with the expression levels of VDAC2 and VDAC3 being lower in most but not all tissues (1, 2). Here, we only briefly consider the VDAC isoforms, as a full chapter in this issue (De pinto et al.) is devoted to VDAC isoforms.

The expression of hVDAC-2 is associated with neurodegenerative diseases, including amylotropic lateral sclerosis (ALS) (5), epilepsy (6), and Alzheimer’s disease (AD) (7). By contrast, VDAC3^−/−^ mice showed only in heart muscle defects in complex IV activity, a component of the electron transfer chain (8).

While both VDAC1- and VDAC3-deficient mice are viable, which permits the study of the role(s) of VDAC in cellular metabolism in intact animals, the reduced number of *Vdac1^−/−^* progeny (according to the Mendelian ratio) suggests partial embryonic lethality (9). Studies using *Vdac^−/−^* mice confirmed the importance of this protein as a transporter of metabolites across the outer mitochondrial membrane (OMM). Detergent-“skinned” muscle fibers, which lack VDAC1, displayed reduced ADP-stimulated oxygen consumption, defects in the electron transport chain (ETC) complex activities, reduction of mitochondria-associated hexokinase (HK), and finally, abnormal mitochondrial morphology (9).

A number of regulatory functions involving the generation of reactive oxygen species (ROS), steroidogenesis, and mitochondria-associated endoplasmic reticulum (ER) pathways have been variously ascribed to the different isoforms (10). VDAC1 is involved in apoptosis, interacting with different proteins and factors and mediating the release of Cyto *c* (1, 11–26). The metabolite transport properties of VDAC1 are also superior to those of VDAC2 and VDAC3 (1). By contrast, VDAC2 is anti-apoptotic (27), is crucial for Bak recruitment (28), and is a critical inhibitor of Bak-mediated apoptosis (29). The anti-tumor agent erastin was found to bind directly to VDAC2 and induce non-apoptotic cell death in some tumor cells that harbored activating mutations in the RAS–RAF–MEK pathway (30).

Immunofluorescence, flow cytometry, and EM immunogold labeling have detected VDAC in other cell compartments in addition to mitochondria (3) [for review, see Ref. (31)]. These compartments include the plasma membrane (3), including location in caveolae and caveolae-like domains (32), the sarcoplasmic reticulum (SR) of skeletal muscles (33), and the ER of rat cerebellum (34). Patch-clamping of intact cells demonstrated channel with properties similar to those of planar-bilayer reconstituted purified VDAC1 (35). VDAC has also been detected in synaptosomes of *Torpedo* electric organ (36). VDAC2 and VDAC3 have been reported in bovine outer dense fibers and in the cytoskeletal component of sperm flagellum (37).

A possible mechanism for targeting VDAC protein to the plasma membrane proposes that the N-terminal signal peptide of the protein is responsible for this targeting (38). Indeed, plasmalemmal (pl) VDAC1 was found to contain a hydrophobic leader sequence (39). Other targeting mechanisms, such as alternative mRNA untranslated regions, were also suggested (35) for trafficking *via* ER/mitochondria-associated membranes or plasma membrane/ER associations (40).

Several possible functions of the extra-mitochondrial VDAC were proposed. These include intracellular communication, as mediating calcium signal between the ER and mitochondria (41), being part of the outwardly rectifying depolarization-induced chloride (ORDIC) channel complex (42), regulate cell volume in brain (43), and mediate ATP release (44). Interestingly, silencing VDAC1 expression by specific small interfering RNA (siRNA) was shown to prevent the entry of amyloid beta (Aβ) into the cytosol, as well as Aβ-induced toxicity (45), suggesting the involvement of pl-VDAC1 in Aβ cell entry and in inducing mitochondrial dysfunction and apoptosis (46). These and other proposed functions for plVDAC were recently presented and discussed (31).

### VDAC1 Structure, Channel Conductance, Properties, and Regulation

The three-dimensional structure of VDAC1 was solved using X-ray crystallography, NMR, and a combination of both (47–49). The methods propose that VDAC1 is composed of 19 β-strands arranged as a barrel, and with the N-terminal domain located within the pore. The pore diameter of the channel has been estimated to be between 3 and 3.8 nm (47) and decreased to about 1.5 nm when the N-terminal domain is located within the pore (47–49).

This imaging-derived structure is in disagreement with the conclusions of biochemical and biophysical approaches, which argue for the existence of additional extra-membranal VDAC1 regions (50). The discrepancy may be attributed to the fact that all three imaging methods employed refolded recombinant *E. coli* expressed VDAC1, purified from inclusion bodies and the refolding conditions may be responsible for the appearance of non-native structures.

An important VDAC1 structural element is the stretch of multiple glycine residues (^21^GlyTyrGlyPheGly^25^) [1,5] that connects the N-terminal domain to β-strand 1 of the barrel. This glycine-rich ^21^GYGFG^25^ sequence, which is highly conserved between mammals, is thought to provide the flexibility required for N-terminal region translocation out of the internal pore of the channel (51). Using site-directed mutagenesis and cysteine substitution, in combination with a thiol-specific cross-linker, it was demonstrated that the N-terminal domain of VDAC1 located within the pore can be translocated (51).

The notion of the mobility of the N-terminal region is further supported by observations that this protein domain moves upon changes in the voltage gradient (51, 52), that antibodies directed against the N-terminal region of the VDAC1 interact with membrane embedded protein (53–55), and that anti-apoptotic and pro-survival factors HK-I, HK-II, and Bcl-2 interact with this domain (16, 51). These results strongly support the suggestion that the N-terminal domain is highly dynamic and can translocate out of the pore (51, 56).

Several different roles have been proposed for the N-terminal segment. These include acting as a voltage sensor (16, 51), as no voltage-dependent conductance was obtained with N-terminal-truncated VDAC1 (16, 57). The N-terminal domain was further proposed to regulate the fluxes of ions and metabolites *via* VDAC1 (49) and to stabilize the β-barrel (58). The structure of the N-terminal domain of VDAC1, its potential role in regulating barrel shape, and its interaction with HK have been reviewed recently (59). Additional roles of this region in VDAC1 oligomerization and regulation of apoptosis (16, 51) are presented below (see VDAC1 Homo-Oligomer Forming the Cyto *c* Release Pathway) and as the binding site for HK, Bcl-2, and Bcl-xL (see VDAC1-Based Peptides As Potential Anti-Cancer Therapy). Thus, it seems that the N-terminal domain of VDAC1 regulates a wide variety of VDAC1 functions, such as its channel conductance and apoptosis.

Another property of VDAC1 is the ability to form an oligomeric structure. Chemical cross-linking and fluorescence resonance energy transfer analysis showed dimers, trimers, tetramers, and higher order oligomers (1, 11, 14, 56, 60–62). In addition, the NMR-based structure of recombinant human (h)VDAC1 implies that it forms a dimer of monomers arranged in parallel (47), while analysis of the crystal packing of murine (m)VDAC1 revealed strong anti-parallel dimers that can further assemble into hexamers (63). The modulation and function of VDAC1 oligomerization are presented below (see VDAC1 Homo-Oligomer Forming the Cyto *c* Release Pathway).

The structural characterization of VDAC2 and VDAC3 is limited, with zebrafish VDAC2 structure was resolved at 2.8A resolution, revealing a dimeric organization (64).

The properties of VDAC1, purified by various procedures and detergents (65), from mitochondria isolated from liver, brain, and other tissues, have been studied when the purified protein was reconstituted into a planar lipid bilayer. Such bilayer-reconstituted VDAC1 assumes a variety of voltage-dependent conformational states, with different selectivities and permeabilities. VDAC1 shows symmetrical bell-shaped voltage-dependent conductance, with the highest conductance (4 nS at 1 M KCl) occurring at low potentials of −20 to +20 mV (1, 66). At low voltages (~10 mV), VDAC1 exists in a high conductive state and shows a preference for transporting anions over cations. VDAC1 is permeable to small ions (e.g., Cl^−^, K^+^, Na^+^), and also to large anions, such as glutamate (66) and ATP (67), and to large cations, such as acetylcholine and dopamine (66). At high positive or negative potentials (>40 mV), VDAC1 switches to lower conductance states with different ionic selectivities and permeabilities (66, 68). In this state, the protein is permeable to small ions but becomes less permeable to ATP and ADP (1, 67, 68).

VDAC1 channel conductance is thought to rely on two separate gating processes, one at positive trans-membrane potentials and the other at negative potentials (1, 68) with the N-terminal α-helical segment of the channel acting as the voltage sensor, gating the pore *via* conformational changes and/or movements (16, 51). Additional studies are required before the VDAC1 gating mechanism can be fully resolved.

### VDAC1 As a Hub Protein

VDAC1 functions in metabolism, Ca^2+^ homeostasis, apoptosis, and other activities are regulated *via* the interaction of VDAC1 with many proteins associated with cell survival and cellular death pathways (1, 14, 56, 61). Indeed, VDAC1 in the OMM serves as a hub protein interacting with diverse sets of cytosolic, ER, and mitochondrial proteins that together regulate metabolic and signaling pathways, providing energy for cellular functions or triggering cell death.

**Figure 1:**
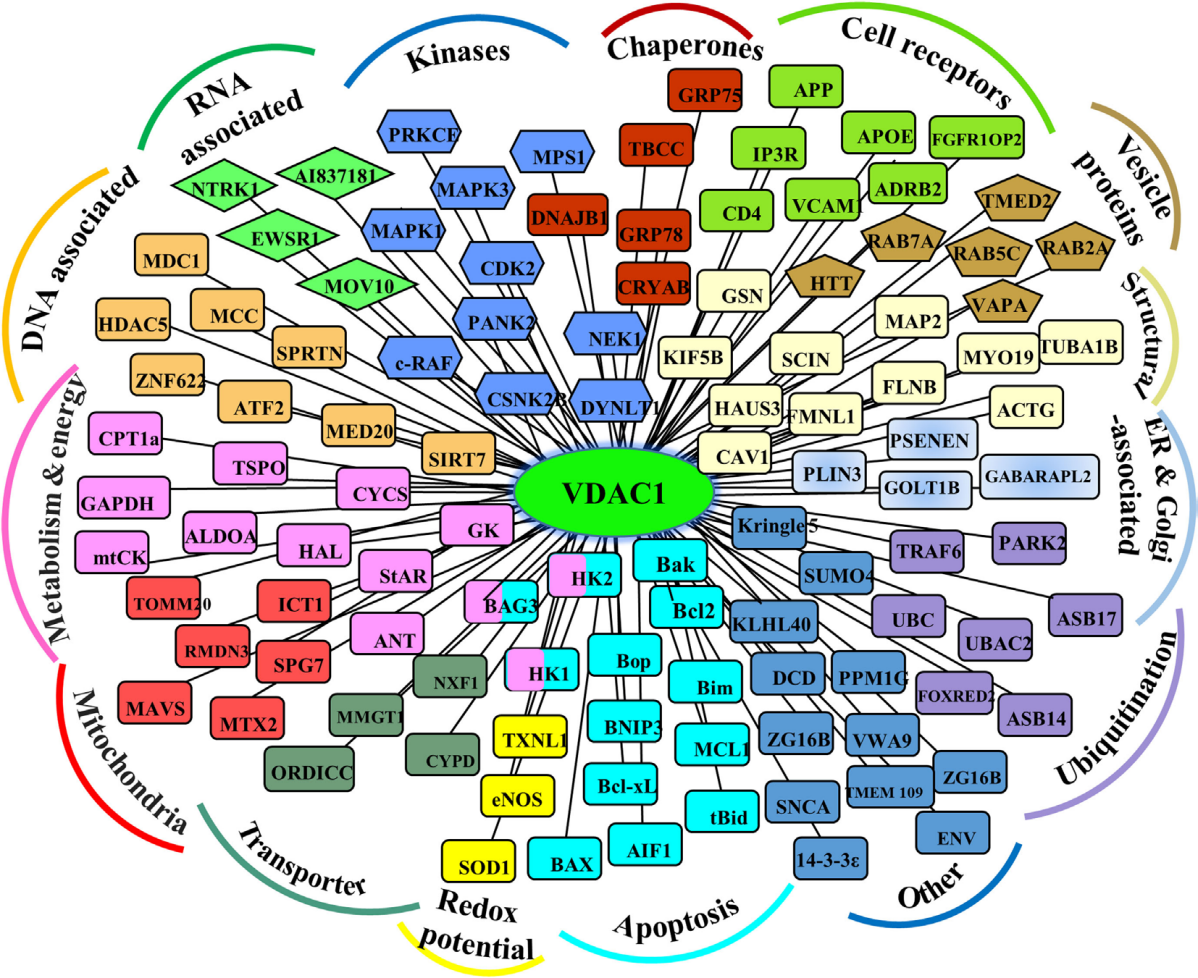
Schematic representation of VDAC1 as a hub protein and its associated proteins. VDAC1 interacting proteins (see Modulation of VDAC1-Mediated Apoptosis and Metabolism *via* Interacting Proteins), sub-grouped into those associated with metabolism, energy, apoptosis, anti-oxidant, cell receptors, or signal transduction, or localized to mitochondria, endoplasmic reticulum (ER), nucleus, and cell membrane.

As support for this viewpoint, the conserved nature of VDAC1 (1), is in agreement with the finding that hub proteins are more evolutionarily conserved than are non-hub proteins (69). The VDAC1 interactome includes proteins involved in metabolism, apoptosis, signal transduction, anti-oxidation, and DNA- and RNA-associated proteins (Figure 1) (1, 11, 14, 56). Furthermore, these proteins may be located in the OMM, inner mitochondrial membrane (IMM), the IMS, the cytosol, ER, plasma membrane, and nucleus. Thus, VDAC1 functions as an anchoring site for proteins that mediate and/or regulate metabolic, apoptotic, and other processes in normal and diseased cells. In addition, some of these proteins, such as Bcl-2, Bcl-xL, and HK, are overexpressed in many cancers (see VDAC Interaction with HK and Other Metabolism-Related Proteins, Interaction of VDAC1 with Bcl-2 Family Members). Importantly, we have been able to develop VDAC1-based peptides, which can interfere with these interactions, leading to impaired cell metabolism and apoptosis (18–20, 70, 71) (see VDAC1-Based Peptides As Potential Anti-Cancer Therapy).

## VDAC1 as a Multi-Substrate Transporter

**Figure 2:**
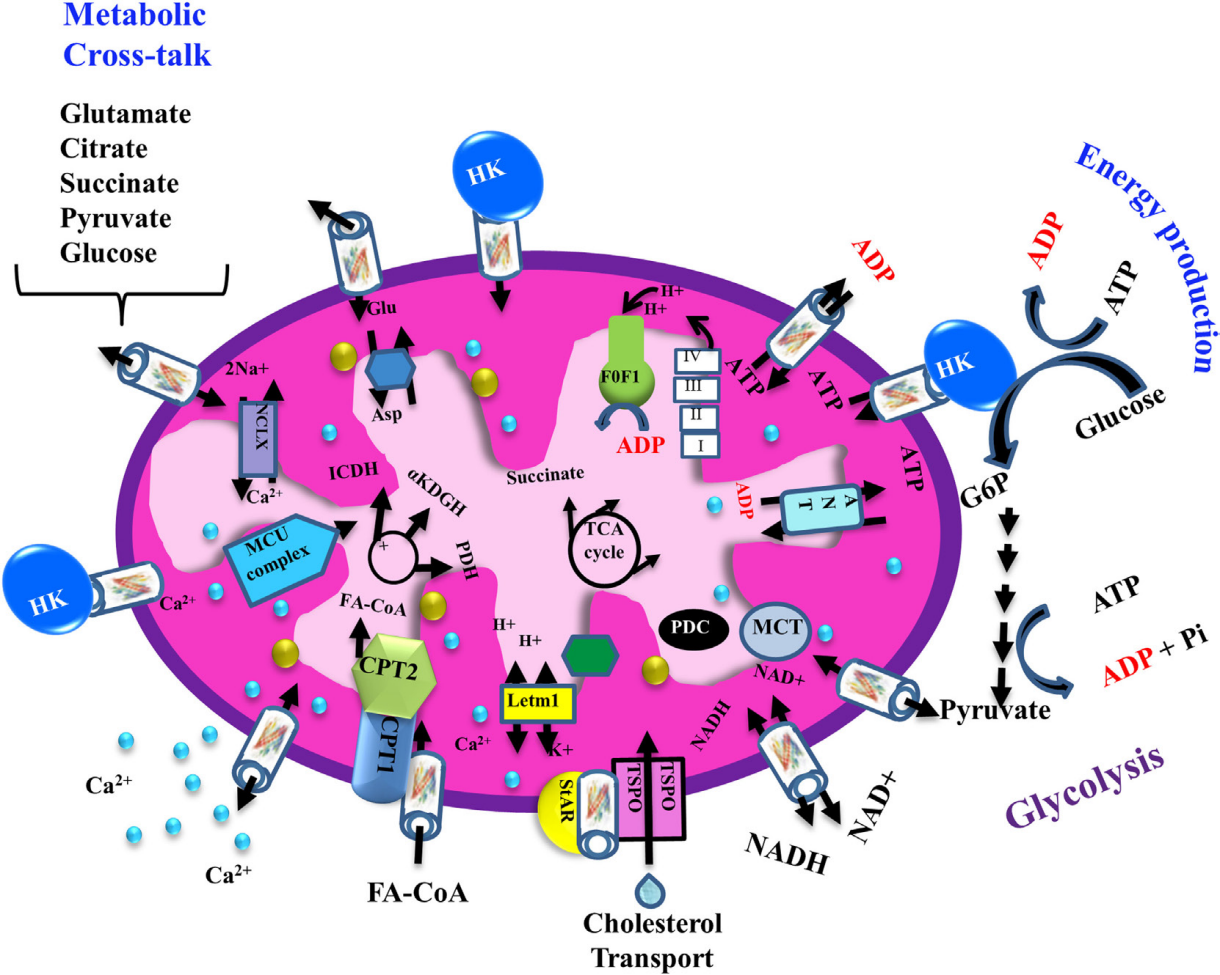
Schematic representation of VDAC1 as a multi-functional channel involved in cell survival and cell death. The various functions of VDAC1 include control of the metabolic cross-talk between the mitochondria and the rest of the cell, cellular energy production [by transporting ATP/ADP and reduced nicotinamide adenine dinucleotide (NADH) between the inter-membrane space and the cytosol and by binding hexokinase (HK)], and Ca^2+^ signaling (by transporting Ca^2+^, acyl-CoAs, and cholesterol); the Ca^2+^ influx and efflux transport systems in the OMMs and IMMs and Ca^2+^-mediated regulation of the tricarboxylic acid (TCA) cycle; activation of pyruvate dehydrogenase (PDH), isocitrate dehydrogenase (ICDH), and α-ketoglutarate dehydrogenase (α KGDH) by intra-mitochondrial Ca^2+^, leading to enhanced activity of the TCA cycle; and control of the electron transport chain and the ATP synthase (F_o_F_1_). VDAC1 in the OMM functions as a Ca^2+^ channel. In the IMM, the uptake of Ca^2+^ into the matrix is mediated by a Ca^2+^-selective transporter, the mitochondrial Ca^2+^ uniporter (MCU), regulated by a calcium-sensing accessory subunit (MICU1). Ca^2+^ efflux is mediated by NCLX, a Na^+^/Ca^2+^ exchanger. The accumulation of high levels of matrix Ca^2+^ triggers the opening of the permeability transition pore (PTP), a fast Ca^2+^ release channel. The role of VDAC1 in the transport of H_2_O_2_ is also presented. In addition, the figure shows the process of transfer of acyl-CoAs across the OMM by VDAC1 to the intermembrane space, where they are converted by carnitine palmitoyltransferase 1a (CPT1a) into acylcarnitine for further process during β-oxidation and cholesterol transport by a multi-protein complex, the transduceosome, containing steroidogenic acute regulatory protein (StAR)/translocator protein (TSPO)/VDAC1. Molecular fluxes are indicated by arrows.

The transport of a variety of metabolites, nucleotides, and coenzymes across the IMM is mediated by about 53 secondary transport proteins called mitochondrial carriers. These transporters are substrate specific, such as the Pi carrier, the ADP/ATP carrier [adenine nucleotide translocase (ANT)] and the aspartate/glutamate carrier (72). On the other hand, VDAC1 is the sole channel mediating the fluxes of ions, nucleotides, and other metabolites up to ~5,000 Da, including hemes and cholesterol, across the OMM (1) (Figure 2). Thus, at the OMM, VDAC1 is perfectly positioned to function as gatekeeper for the entry and exit of substrates and products into and out of the mitochondria, and to interact with proteins that mediate and regulate the integration of mitochondrial functions with other cellular activities (1, 12, 14, 56, 61, 68, 73) (Figures 1 and 2).

### VDAC1 As a Cellular Metabolite Transporter Controls Cell Energy and Metabolites

VDAC1 mediates the passage of metabolites, including pyruvate, malate, and succinate, into and out of mitochondria (1). In addition, VDAC1 also allows shuttling of ATP and ADP, and NAD+/reduced nicotinamide adenine dinucleotide, with mitochondria-generated ATP being transported to the cytosol in exchange for ADP, which is utilized in oxidative phosphorylation (OXPHOS) to generate ATP. As such, VDAC1 controls the ETC (1) (Figure 2) as well as the normal flow of metabolites (74). The importance of VDAC1 in cell energy and metabolism homeostasis is reflected in the findings that closure of VDAC1 (73), or downregulation of VDAC1 expression decreased metabolite exchange between mitochondria and the cytosol and inhibited cell growth (53, 75) [see Silencing VDAC1 Expression by Short Hairpin RNA (shRNA) or siRNA as a Tool to Reprogram Cancer Cell Metabolism].

The importance of VDAC1 in channeling ATP from the mitochondria to kinases has been presented in several studies. These showed that VDAC1 interacts with HK and creatine kinase (CrK) that produce high-energy metabolites, such as glucose-6-phosphate (G-6-P) and creatine phosphate in brain and muscle, respectively. The interaction of VDAC1 with HK mediates a coupling between OXPHOS and glycolysis (see Alterations in VDAC1 Expression Level in Cancer), while VDAC1 forms a complex with the ANT, and CrK at the contact sites between the IMM and OMM (76). Dimeric αβ-tubulin was proposed as a regulator of permeability of VDAC1 to ATP, with monomers of αβ-tubulin decreasing the passage of ATP through the channel (77). The function of VDAC1 in energy metabolism of cancer cells and the significance of the overexpression in many cancer cells (11) is discussed further below (see VDAC1 Expression Level and Cell Death Induction—a New Concept, Unraveling VDAC1-Based Therapies).

Cholesterol is another metabolite transported across the OMM (78) (Figure 2), with VDAC1 being a component of a multi-protein complex, the transduceosome, involved in the process. In addition to VDAC1, the transduceosome also includes the OMM high-affinity cholesterol-binding protein translocator protein (TSPO) and the steroidogenic acute regulatory protein (79) (Figure 2).

Cholesterol synthesis is highly elevated in various cancer cells, mainly in the OMM (80). In cancer cells, the increased mitochondria-bound HK is proposed to increase synthesis and uptake of cholesterol into the mitochondria. (81). Recently, it has been suggested that a glycine rich motif ^21^GYGFG^25^ sequence in the N-terminal part of VDAC1 is responsible for cholesterol binding (82). Cholesterol at high levels can reduce the activity of membrane-associated proteins and, thus, inhibit the metabolic functions of VDAC1 (83).

Thus, VDAC1 is involved in cholesterol synthesis and transport, and is regulated by cholesterol.

Finally, in rat liver mitochondria, VDAC1 is proposed as part of a complex mediating the transport of fatty acids through the OMM (84). In this case, the hypothesis is that VDAC1 allows docking of the long-chain acyl-CoA synthetase (ACSL) at the OMM and, thus, linking it to carnitine palmitoyltransferase 1a (CPT1a). According to this proposal, activation of VDAC1 by ACSL allows the transfers acyl-CoAs *via* VDAC1, thus acrossing the OMM to the IMS, where they are converted into acylcarnitine by CPT1a. Finally, it was recently proposed that VDAC serves as a lipid sensor (85).

### VDAC1 As a Ca^2+^ and ROS Transporter

**Figure 3:**
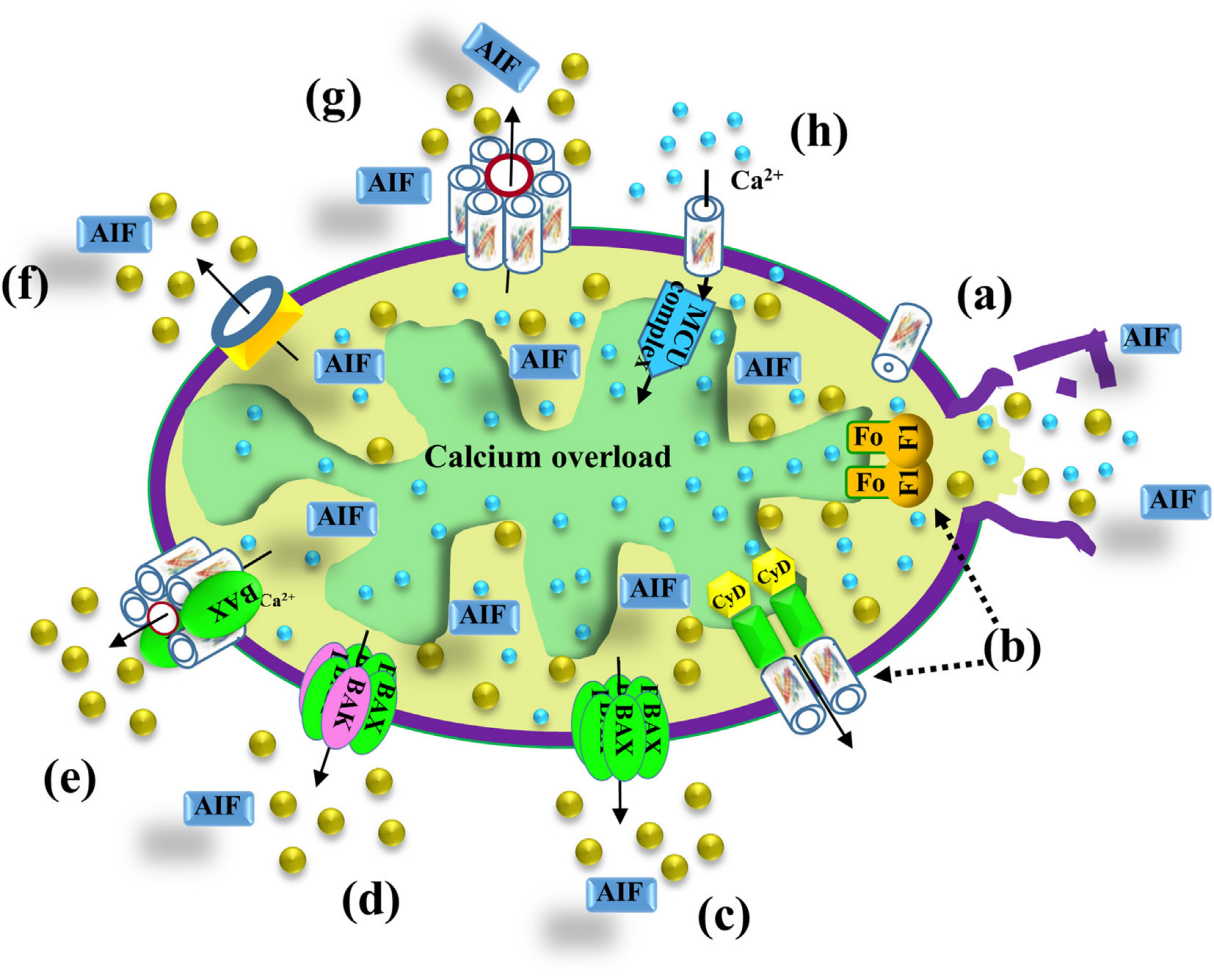
VDAC1 function in cell death. Different models proposed for the release of apoptogenic proteins, such as Cyto *c* (gold balls) and apoptosis-inducing factor (AIF), from the mitochondrial inter-membrane space to the cytosol, leading to apoptosis. These models include (*a*) *VDAC1 closure and outer mitochondrial membrane (OMM) rupture serving as the Cyto c release pathway*—prolonged VDAC1 closure leads to mitochondrial matrix swelling and OMM rupture, resulting in the appearance of a non-specific release pathway for apoptogenic proteins; (*b*) *a permeability transition pore (PTP) provides the apoptogenic protein release pathway*—a large conductance pore-forming complex, the PTP, composed of VDAC1 in the OMM, adenine nucleotide translocase in the inner mitochondrial membrane (IMM), and cyclophilin D (CyD) in the matrix, allows apoptogenic protein release; (*c*) *Bax activation, followed by its oligomerization, results in OMM permeabilization*—upon apoptosis induction, Bax becomes associated with mitochondria as a large oligomer/complex forming a Cyto *c*-conducting channel in the OMM; (*d*) *a pore is formed by oligomerized forms of Bax and Bak*—Bax/Bak oligomerization, supposedly activated by BH3-only proteins (e.g., Bid), results in OMM permeabilization and Cyto *c* release; (*e*) *a Bax- and VDAC1-based hetro-oligomer mediates Cyto c release*—the interaction of pro-apoptotic proteins (Bax/Bak) with VDAC1 forms a cytochrome *c* (Cyto *c*) release pathway; (*f*) *mitochondrial apoptosis-inducing channel (MAC) as the release pathway*—MAC offers a high-conductance channel and a putative Cyto *c* release channel; (*g*) *oligomeric VDAC1 as a channel for the release of apoptotic proteins*—a protein-conducting channel is formed within a VDAC1 homo-oligomer, allowing Cyto *c* release and apoptotic cell death; (*h*) *mitochondrial Ca^2+^ overload induces apoptosis*—following Ca^2+^ overload in the matrix, Ca^2+^ transport mediated by VDAC1 across the OMM and by the mitochondrial Ca^2+^ uniporter (MCU) in the IMM leads to dissipation of the membrane potential, mitochondria swelling, PTP opening, Cyto *c* release, and apoptotic cell death. PTP opening is also accompanied by an efflux of the accumulated Ca^2+^ into the cytosol.

Mitochondria are also a major hub of cellular Ca^2+^ homeostasis that is fundamental for a wide range of cellular activities. Intra-mitochondrial Ca^2+^ controls energy metabolism modulation of critical enzymes, such as members of the tricarboxylic acid (TCA) cycle and enzymes responsible for fatty acid oxidation (FAO) (86) (Figure 2). Ca^2+^ overload in the mitochondria is involved in apoptotic cell death, triggering Cyto *c* release, and subsequent cell death (Figure 3).

Ca^2+^ transport across the IMM is mediated by several proteins, including the mitochondrial Ca^2+^ uniporter (87, 88) and the Ca^2+^ efflux mediator Na^+^/Ca^2+^ exchanger NCLX (89, 90) (Figure 2). A number of studies have reported that VDAC1 in the OMM can transport Ca^2+^ (91–94). VDAC1 possesses divalent cation-binding site(s) (91, 95) and it was proposed that VDAC1 activity is modulated by physiological [Ca^2+^]i (94) with the magnitude of transfer into the mitochondrial matrix regulated by Bcl-xL binding to VDAC (96, 97). VDAC1 also functions in the Ca^2+^ cross-talk between ER and mitochondria (98).

Inhibitors of VDAC1, such as 4,4′-diisothiocyanostilbene-2,2′-disulfonic acid (DIDS), were shown to prevent the apoptosis stimulus following an increase in intracellular Ca^2+^ levels (99) or Ca^2+^-mediated oxidative stress and apoptosis, as induced by 5-aminolevulinic acid (100).

Mitochondria are a major source of ROS, that are linked to anti-tumor immunity, the tumor microenvironment, proliferation, and death of cancer cells (101). While ROS promote tumorigenicity through signaling, they can also selectively kill a number of cancer cell lines (102) as well as normal cells, attacking DNA, lipids, and proteins (103). VDAC1 has been proposed to mediate ROS release from the IMS to the cytosol (104) (Figure 2), with HK-I and HK-II bound to VDAC1 decreasing this release (105), and thereby reducing intracellular levels of ROS (106).

## Cancer, Metabolism, Mitochondria, and VDAC1

It is now well accepted that in cancers, regardless of cellular or tissue origin, impaired cellular energy metabolism is the defining characteristic. Cancer cells exhibit significant metabolic alterations with respect to several critical substrates, including important changes in the metabolism of both glucose and glutamine that require plasticity of the metabolic machinery (107, 108). The view of cancer as a metabolic disease originated with the experiments of Otto Warburg in the 1920s, however, this view was gradually displaced by the concept of cancer as a genetic disease. Recently, although the Warburg effect and metabolic defects expressed in cancer cells are thought to arise primarily from genomic mutability selected during tumor progression (109, 110), the genetic origin of cancer has come into question. Accordingly, the genomic instability and essentially all hallmarks of cancer, including aerobic glycolysis were connected to mitochondria dysfunction and energy metabolism (111, 112). Indeed, supporting evidence suggests that cancer is primarily a mitochondrial metabolic disease (111).

Evidence for a metabolic rather than genetic origin includes the absence of a specific gene mutation or chromosomal abnormality that is common to all cancers (113), while nearly all cancers display aerobic glycolysis, regardless of their tissue or cellular origin. Cancer cells typically display high rates of glycolysis, even when fully oxygenated (aerobic glycolysis), and an altered redox balance (Warburg effect) (114–116). To increase glycolysis, cancer cells upregulate the transcription of genes involved in the glycolytic pathway (i.e., glucose transporters, glycolytic enzymes, etc.). Cancer cells actually use both glycolysis and OXPHOS, according to normoxic or hypoxic conditions and their capacity to regulate the expression of genes important for cell growth (117). By regulating the metabolic and energetic functions of mitochondria, VDAC1 can, therefore, control the fate of cancer cells. Mitochondrial-bound HK, considered the rate-limiting enzyme of glycolysis, is overexpressed in cancer (1, 23, 118) and, as discussed in Section “VDAC Interaction with HK and Other Metabolism-Related Proteins,” is associated with VDAC1, an interaction that offers several advantages to cancer cells (1, 11), as outlined below (see VDAC Interaction with HK and Other Metabolism-Related Proteins). The HK–VDAC1 complex formation is regulated by Akt (119) and glycogen synthase kinase 3 beta (GSK3β), while the HK–VDAC1 complex is disrupted by VDAC1 phosphorylation (24). In addition, it was shown that an increase in the amount of free cytoplasmic tubulin decreased VDAC conductance and mitochondrial membrane potential (ΔΨm) in all three VDAC isoforms (120).

The association of VDAC1 with the process of FAO is also important. Hetero-oligomeric complexes containing VDAC and CPT1a, a liver OMM protein catalyzing the first step in mitochondrial FAO and ACSL were detected and thought to transfer activated fatty acids through the OMM (84).

Specifically targeting metabolism in cancer cells presents a potential therapeutic strategy. However, although glucose metabolism is increased in cancer cells, they mostly use the same glycolytic enzymes as do normal cells so that the choice of glycolytic enzymes as a target for cancer treatment may increase the risk of affecting normal cells as well (121).

## Mitochondria, Apoptosis, VDAC, and Cancer

Apoptosis can be initiated by two signaling cascades, the extrinsic and intrinsic pathways. The extrinsic pathway can be activated by binding of tumor necrosis factor (TNF), tumor necrosis factor-related apoptosis-inducing ligand (TRAIL), and other ligands to their specific death receptor (122). Such interactions lead to cascade of events leading to activation of procaspase 8 which then activates cysteinyl/aspartate-specific protease (caspases)-3, -7, and -6, promoting apoptosis (123).

The intrinsic pathway can be activated by intracellular signals, such as oxidative stress, Ca^2+^ overload, DNA damage, and by various compounds, such as chemotherapeutic drugs (124). This leads to the release of IMS apoptogenic proteins [e.g., Cyto *c*, apoptosis-inducing factor (AIF), Smac/DIABLO] (124). The released Cyto *c* is a constituent of the apoptosome and activates procaspase-9, which in turn, activates the executioner caspases, caspase-3 and -7, leading to cell destruction (124). The AIF released is translocated to the nucleus, leading to chromatin condensation and DNA fragmentation (124). The intrinsic pathway is regulated by members of the B-cell lymphoma 2 (Bcl-2) family of proteins (125), and by the inhibitor of apoptosis protein (IAP) family of proteins (126).

The two pathways can be connected through the caspase-8-mediated cleavage of Bid into tBid (truncated Bid), which is translocated to the mitochondria, where it causes Cyto *c* release and subsequent cell death (123). In this way, tBid links extrinsic pathway to intrinsic, mitochondria-mediated apoptosis.

### Cancer Cells Avoid Apoptosis

In many cancers, there is a deregulation of the balance between cell growth and death (115). Tumor cells avoid apoptosis by alterations in the expression levels of pro- and anti-apoptotic proteins, as well as because of reduced caspase function and impaired death receptor signaling (127). Overexpression of anti-apoptotic proteins, such as Bcl-2 and Bcl-xL, has been demonstrated in numerous cancers, including colon, thyroid, breast, and endometrial cancer (128). Moreover, Bcl-2 expression is correlated with the degree of aggressiveness and resistance to chemotherapy-induced apoptosis (129).

One of the most common apoptotic pathways involves transcription factor p53, which plays a role in promoting transcription of pro-apoptotic factors, such as Puma, Noxa, Bax, and apoptosis protease-activating factor 1 (APAF1). In response to a spectrum of apoptotic stimuli, such as oxidative stress, p53 translocates to the mitochondria (130), where it may regulate VDAC1-mediated apoptosis (131). As already discussed, cancer cells possess elevated levels of mitochondria-bound HK that not only enhances glycolysis but also protects against mitochondria-mediated apoptosis *via* direct interaction with VDAC1 (16, 17, 20, 21, 132, 133).

Although induction of apoptosis in cancer cells conceptually represents an effective therapeutic approach, conventional apoptosis-inducing chemotherapy is limited by a lack of specificity, by cancer cell resistance, and by side effects of cytotoxicity for normal cells.

### VDAC1 Involvement in Apoptosis

VDAC1 is now well-accepted as an important player in apoptosis and is being explored as a new target for cancer therapy (1, 11, 13–15, 55, 134). Evidence supporting the activity of VDAC1 in apoptosis includes (a) Cyto *c* release, cell death, and Bax–VDAC1 interaction were all inhibited by anti-VDAC1 antibodies (135–137); (b) HK-I and HK-II interacting with VDAC1 inhibited staurosporine (STS)-induced Cyto *c* release and apoptosis in native but not mutated VDAC1-expressing cells (14, 17, 133); (c) the interaction of ruthenium red (RuR) with native but not mutated VDAC1 prevented Cyto *c* release and apoptosis (95, 133, 138); (d) siRNA-mediated downregulation of VDAC1 prevented cell death induced by cisplatin (139); (e) reducing the level of VDAC1 expression attenuated endostatin (ES)-induced apoptosis (140); (f) over-expression of VDAC1 induced apoptosis, regardless of cell type and the effect was antagonized by anti-apoptotic proteins (17, 133, 141); (g) VDAC1 mediated Cyto *c* release from proteoliposomes (25, 62, 135); (h) VDAC1-deficient mitochondria from mutant yeast did not exhibit Bax/Bak-induced Cyto *c* release (25, 142); (i) the anti-apoptotic effect of Bcl-2 and Bcl-xL was obtained in cells expressing native but not mutated VDAC1 (18, 19); (j) VDAC1 channel conductance inhibitors, such as DIDS, DPC (diphenylamine-2-carboxylate), and VBIT-4, inhibited apoptosis triggered by various inducers (99, 104, 133, 143); and finally, (k) cyathin-R, a cyathane-type diterpenoid from the medicinal fungus *Cyathus africanus*, could induce apoptosis in Bax/Bak-depleted cells but not when VDAC1 was depleted. Cyathin-R-induced apoptosis was inhibited by DPC (144).

### Proposed Pathways and Mechanisms for Apoptogenic Factor Release from Mitochondria

All of the mitochondrial apoptotic proteins (Cyto *c*, AIF, Smac/DIABLO, and endonuclease G) that activate apoptosis in the cytosol are located in the IMS. Therefore, only increase in the OMM permeability is required for the release of apoptogenic proteins. It remains, however, unclear how these pro-apoptotic proteins cross the OMM for release into the cytosol. Based on a variety of approaches and strategies, several mechanisms describing the release of apoptotic proteins from the IMS were proposed [for reviews, see Ref. (1, 11–13, 61, 145)] (Figure 3). Some models propose that release of apoptotic proteins from the IMS is facilitated by a swelling of the mitochondrial matrix and subsequent rupture of OMM integrity (Figure 3a). Other models predict the formation of large channels that can allow the passage of Cyto *c* and other proteins and, thus, to be released from the IMS to the cytosol (Figure 3) such as:

(i) *Permeability transition pore (PTP)*: the mitochondrial PTP is a high-conductance non-specific pore activated by ROS, Ca^2+^ overload, and other agents, leading to mitochondrial swelling and the release of Cyto *c* into the cytosol. Initially, PTP was proposed to comprising VDAC1 in the OMM, ANT in the IMM, and cyclophilin D (CyD), a resident of the matrix (55, 146, 147) (Figure 3b,h). However, in knockout experiments performed in mice, even mitochondria examined from cells lacking some but not all ANT isoforms (148, 149), or VDAC1 (150) showed PTP formation. Recently, it was proposed that dimers of the ATP synthase complex can form the PTP (151).
(ii) *Bax/Bak complexes*: Bax and Bak are pro-apoptotic proteins proposed to oligomerize to form a Cyto *c* release channel (152–154) (Figure 3c,d). Bcl-2 prevents Bax oligomerization and insertion into the OMM (152, 153). Bak can form a large pore due to its oligomerization (155) or due to formation of hetro-oligomers with Bax following their activation by tBid (156, 157).
(iii) *Bax/VDAC1 complexes*: VDAC1 and Bax form hetero-oligomers forming the Cyto *c* release channel (26, 158) (Figure 3e).
(iv) *Mitochondrial apoptosis-inducing channel (MAC*): MAC a supra-molecular complex forming channel at the OMM that can mediate Cyto *c* release (159) (Figure 3f). Recently, it was proposed that Bax and/or Bak form the MAC (160).
(v) *VDAC1 oligomerization*: upon apoptosis induction, VDAC1 undergoes conformational changes and oligomerization, forming a channel within the homo-oligomer large enough to allow Cyto *c* release, and subsequent apoptosis (14, 16, 56, 60–62, 145, 161, 162) (Figure 3g) (see VDAC1 Homo-Oligomer Forming the Cyto *c* Release Pathway). It was also suggested that apoptosis stimuli lead to VDAC1 oligomerization by inducing upregulation of VDAC1 expression levels (see VDAC1 Expression Level and Cell Death Induction—a New Concept).

Finally, it should be noted that the release of Cyto *c* could be achieved by either of the above proposed mechanisms, depending on the nature of the apoptosis inducer and cell type (163).

### VDAC1 Homo-Oligomer Forming the Cyto *c* Release Pathway

The determined VDAC1 pore diameter in its high conductance state is about 2.6–3.0 nm. This diameter is too small to allow the passage of Cyto *c* to be released to the cytosol. To overcome this issue, the formation of a large channel comprising VDAC1 monomers has been proposed to serve as the Cyto *c* release channel (56, 60–62, 145, 161, 162). VDAC1 is known to exist as higher-order oligomers (22, 60, 62, 145, 164–166) (see VDAC1 Structure, Channel Conductance, Properties, and Regulation) and both purified soluble and membrane-embedded VDAC1 have been shown to assemble into dimers, trimers, and tetramers in a dynamic process (62). Recently, the particular lipid composition of the OMM has been shown to significantly enhance VDAC1 oligomerization (167). Finally, the connection between VDAC1 oligomerization and Cyto *c* release was supported by the finding that VDAC1 oligomerization was highly enhanced by various inducers of apoptosis, including STS, curcumin, arsenic trioxide (As_2_O_3_), etoposide, cisplatin, selenite, TNF-α, H_2_O_2_, or UV light (60) reflecting a shift in VDAC1 status from the monomeric toward the oligomeric form. The oligomerization of VDAC1 was favored regardless of cell type or mechanism of action of the apoptosis inducer used, although all affected mitochondria. In addition, As_2_O_3_-induced homo-dimerization of VDAC1 was prevented by overexpression of the anti-apoptotic protein, Bcl-xL (137). The association between apoptosis induction and VDAC1 oligomerization gained support with the finding that both processes are inhibited by DIDS, SITS (4-acetamido-4′-isothiocyanato-stilbene-2,2′-disulfonic acid), H_2_DIDS (4,4′-diisothiocyanatodihydrostilbene-2,2′-disulfonic acid), DNDS (4,4′-dinitrostilbene-2,2′-disulfonic acid), and DPC, known anion transport inhibitors that all interact with VDAC1 (60, 99, 144, 145, 168).

**Figure 4:**
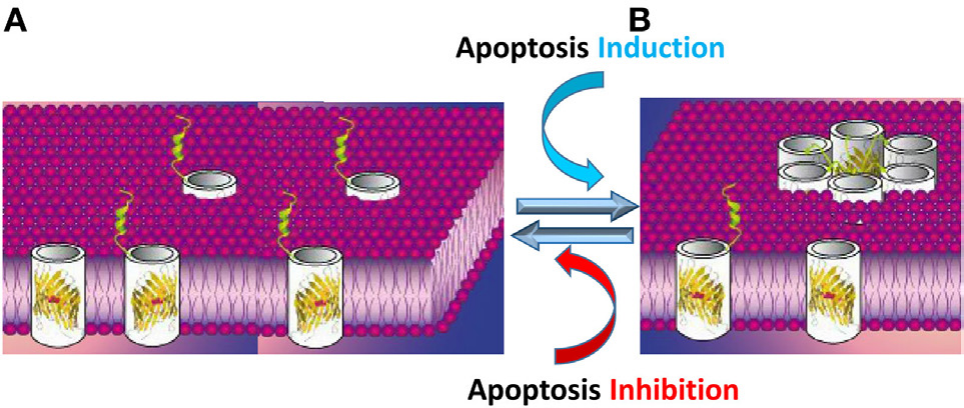
Apoptosis stimuli induce VDAC1 oligomerization: a proposed Cyto *c* release channel A. Before apoptosis induction, VDAC1 is in the monomeric state, with the amphipathic α-helix N-terminal region cytoplasmically exposed (169) or positioned within the pore (47–49). **(B)** Upon apoptotic signaling, VDAC1 oligomerizes to form a multimer and the amphipathic α-helix N-terminal region of each VDAC1 molecule flips into the hydrophobic pore formed by the β-barrels, forming a hydrophilic pore capable of conducting Cyto *c*.

The results presented above led us to propose a novel model in which VDAC1 exists in a dynamic equilibrium between the monomeric and oligomeric states, with apoptosis inducers shifting the equilibrium toward oligomers. VDAC1 oligomers form a large flexible pore between individual subunits of VDAC1, mediating the passage of released Cyto *c* across the OMM, leading to cell death (14, 56, 60–62, 144, 145, 161, 162) (Figure 4).

The passage of Cyto *c* requires a channel formed by minimal number of six VDAC1 monomers. When arranged in a circle, the central pore should be 4.0 nm in diameter, allowing the transport of Cyto *c* (3.4 nm external diameter). The formation of VDAC1 hexamers and higher oligomers involves the formation of several contact sites between adjacent VDAC1 monomers, involving β-strands 1, 2, 18, and 19 from both VDAC1 monomers (47, 63).

The contact sites between VDAC1 molecules in dimers and higher oligomers were identified using structural- and computational-based approaches, in combination with site-directed mutagenesis, cysteine replacement and chemical cross-linking (170). A contact site involving β-strands 1, 2, and 19 that undergoes conformational changes following apoptosis induction and assembles into higher oligomeric states with contact sites involving β-strands 8 and 16 was identified in dimeric VDAC1 (170).

The proposed VDAC1 channel formed upon its oligomerization is composed of several β-barrels; thus, is expected to be hydrophobic, making it difficult for the positively charged Cyto *c* to cross such a pore. As discussed above (see VDAC1 As a Hub Protein), structural, biochemical, and functional studies revealed that the N-terminal domain is mobile and can be cytoplasmically exposed. Following apoptosis induction along with VDAC1 oligomerization, the amphipathic N-terminal segment is thought to be move out of the pore and interact with surface of the newly formed channel in the oligomeric structure (51, 56, 168) (Figure 4). The N-terminal domain is relocated in close proximity to Glu72 and is also surrounded by other hydrophilic residues, thereby converting the hydrophobic pore into a hydrophilic pore, allowing the passage of Cyto *c* and other apoptogenic proteins across the OMM (16, 51, 56, 60, 168). Importantly, the N-terminal domain is a target for anti-apoptotic proteins, interacting with HK, Bcl-2, and Bcl-xL (16, 18, 19, 51). Thus, anti-apoptotic proteins may function by interacting with the VDAC1 N-terminal domain and preventing the conversion of the pore to a hydrophilic environment and, hence, the translocation of apoptogenic proteins from the IMS to the cytosol to trigger apoptosis.

## VDAC1 Expression Level and Cell Death Induction—A New Concept

### Exogenous VDAC1 Overexpression Induces Cell Death

The expression level of VDAC1 plays a critical role in mitochondria-mediated apoptosis, as demonstrated using silencing or overexpression approaches (1, 16, 17, 133, 141, 161, 165, 171, 172). VDAC1 overexpression-induced apoptosis was inhibited by VDAC1-interacted compounds RuR (133, 138), Bcl-2, and DIDS (171), or by overexpression of HK-I (17, 21, 133). As further presented below (see Apoptosis Stimuli Induce VDAC1 Overexpression in Ca^2+^-Dependent Manner, Proposed Mechanism for Cell Death Induction by VDAC1 Overexpression—A New Concept), the cellular expression level of VDAC1, associated with its oligomerization is an important factor regulating mitochondria-mediated apoptosis.

### Apoptosis Stimuli Induce VDAC1 Overexpression in Ca^2+^-Dependent Manner

Several studies have demonstrated that the induction of apoptosis is followed by an increase in the levels of VDAC1 expression (12), UV irradiation (173), ROS (174), and arsenic trioxide (As_2_O_3_) (175).

Other examples include arbutin (hydroquinone-*O*-beta-d-glucopyranoside), a tyrosinase inhibitor in A375 human malignant melanoma cells (176), and somatostatin, used in the treatment of advanced prostate cancer, which upregulated the expression of VDAC1 and VDAC2 in the LNCaP prostate cancer cell line (177). Upregulation of VDAC1 expression was noted in acute lymphoblastic leukemia cell lines following prednisolone treatment (178). Cisplatin-induced VDAC1 overexpression in a cisplatin-sensitive cervix squamous cell carcinoma cell line (A431) but downregulation VDAC1 in a cisplatin-resistant cell line (A431/Pt) (179).

The causal nature of the relationship between VDAC1 expression levels and sensitivity to various treatments was illustrated in several studies (12, 161) where the correlation with drug efficacy suggests that numerous anti-cancer drugs and treatments act *via* the regulation of VDAC1 expression.

Apoptosis induction was shown to disrupt [Ca^2+^]i homeostasis and energy production (180). Indeed, many anti-cancer drugs and other cytotoxic agents, such as thapsigargin, STS, As_2_O_3_, and selenite, induce apoptosis, and disrupt [Ca^2+^]i homeostasis (161, 162). The mechanism of upregulation of VDAC1 by the apoptosis induction is thought to involve an increase in [Ca^2+^]i (161, 162). The overexpressed VDAC1 then forms oligomers (as discussed in Section “VDAC1 Homo-Oligomer Forming the Cyto *c* Release Pathway”) and this triggers Cyto *c* release and, finally, cell death (161, 162). In support of this proposal, AKOS-022 and VBIT-4, compounds that interact directly with VDAC1 to prevent oligomerization, prevented the process of elevation of [Ca^2+^]i, Cyto *c* release, and apoptosis (143).

Thus, although mechanisms employed by apoptosis inducers may differ, they all induce VDAC1 overexpression in a Ca^2+^-dependent manner (161), suggesting that elevation [Ca^2+^] and upregulation of VDAC1 represent a common mechanism of apoptotic stimuli.

### Proposed Mechanism for Cell Death Induction by VDAC1 Overexpression—A New Concept

Upregulation of the expression of VDAC1 may, therefore, represent a new common denominator for the mechanism of action of apoptosis. This proposal is based on the observation that several cancer drugs and treatments induce apoptosis as well as upregulating VDAC1 expression levels (176–179, 181).

Accordingly, a new concept of apoptosis induction can be formulated as: apoptosis *inducers ⟶ enhanced VDAC1 expression levels ⟶ VDAC1 oligomerization ⟶ Cyto c release ⟶ apoptosis*.

This novel mechanism provides a platform for developing a new class of drugs to treat cancer acting *via* modulating VDAC1 levels through action on the gene promoter.

## Modulation of VDAC1-Mediated Apoptosis by Small Molecules Acting *via* Direct Interaction with VDAC1

Several compounds have been shown to interact with VDAC1 and thereby modulate the protein’s apoptotic function either positively or negatively. Some examples are given below.

### Molecules Interacting with VDAC to Induce Apoptosis

*Erastin* is an anti-tumor agent selective for tumor cells bearing oncogenic RAS (30). The compound binds VDAC2 but not N-terminal truncated hVDAC2 (182).

*Oblimersen (G3139)*, an 18-mer phosphorothioate anti-sense oligonucleotide targeting the initiation codon region of Bcl-2 mRNA, has been shown to bind to bilayer-reconstituted VDAC1 and reduces the channel conductance (183).

*Avicins* represent a family of triterpenoid saponins, which exhibit cytotoxic activity in tumor cells, as well as anti-inflammatory and anti-oxidant properties. Avicins interact with bilayer-reconstituted VDAC1 to reduce its channel conductance (184) and permeabilize the OMM to induce Cyto *c* release (185).

*Cisplatin* is a widely used anti-cancer drug that acts by inducing apoptosis through the formation of inter- and intra-strand nuclear DNA cross-links. Mitochondria have also been implicated as a cisplatin target. Cisplatin binds to VDAC1 (186) and modulates VDAC1 activity (179). It has been suggested that VDAC1 may serve as a cisplatin receptor in apoptotic pathways (187).

*Endostatin* has been shown to promote PTP opening *via* binding to VDAC1. Silencing VDAC1 expression by siRNA attenuated ES-induced apoptosis in endothelial cells (140).

*Methyl jasmonate* (*MJ*) is a natural cyclopentanone lipid belonging to the jasmonate family of plant oxylipin stress hormones (188). MJ interacts directly with VDAC1 to reduce the channel conductance and also detaches HK from VDAC1 (189).

*Cyathin-R*, a cyathane diterpenoid, was found to interact with VDAC1 to decrease channel conductance and induce VDAC1 oligomerization and apoptosis in Bax/Bak-deficient cells (144). Cyathin-R-induced VDAC1 oligomerization and apoptosis were inhibited by VDAC1-interacting molecules, such as DIDS, SITS, DNDS, and DPC (144). si-RNA targeting VDAC1 to reduce its expression prevented cyathin-R-induced apoptosis. Moreover, cyathin-R effectively attenuated tumor growth when Bax/Bak-deficient cells were implanted into a xenograft mouse model (144). Cyathin-R, thus, represents a potential lead candidate to be an effective anti-cancer drug, inducing cell death in cancerous cells with inactivated Bax/Bak.

### Molecules Interacting with VDAC1 to Inhibit Apoptosis

*DIDS, SITS, H2DIDS, DNDS*, and *DPC*, which are known anion transport inhibitors, all interact with VDAC1, and inhibit apoptosis stimuli-induced apoptosis and VDAC1 oligomerization in many cancer cell lines (99, 144). DIDS blocked apoptosis triggered by overexpression of rice VDAC in mammalian cells (171) and prevented cisplatin-(139) and STS-induced (190) cell death.

*AKOS-022 and VBIT-4* are newly developed apoptosis inhibitors whose direct interaction with VDAC1 reduces VDAC1 channel conductance and prevents VDAC1 oligomerization and apoptosis in a number of cell lines (143).

*Ruthenium red* (55, 91) and the *ruthenium amine binuclear complex* (Ru360) (191), which are proposed to specifically interact with Ca^2+^-binding proteins, decreased the channel conductance of VDAC1. RuR protects against cell death induced by various stimuli (91, 192), or by VDAC1 overexpression (133). RuR had no effect on cells expressing E72Q-mVDAC1, or on VDAC1 channel conductance, suggesting that RuR-mediated protection against apoptosis is exerted through its direct interaction with VDAC1 (138).

The action of these apoptosis inhibitors thus supports the concept of a tight coupling between VDAC1 oligomerization and apoptosis induction. Inhibiting apoptosis at an early stage, such as VDAC1 oligomerization, may be an effective approach to block or slow apoptosis in neurodegenerative disorders (193) and various cardiovascular diseases, where there is enhanced apoptosis (194, 195).

## Modulation of VDAC1-Mediated Apoptosis and Metabolism *via* Interacting Proteins

The location of VDAC1 in the OMM positions it well to interact with proteins that mediate and regulate the integration of mitochondrial functions with other cellular activities (Figure 1). Here, we focus on the interactions of VDAC1 with proteins associated with cancer.

### VDAC Interaction with HK and Other Metabolism-Related Proteins

The multiple regulatory roles of VDAC1 in cell metabolism are mediated not only through its function in the energy production and metabolic cross-talk between the mitochondria and the rest of the cell but also *via* interactions with other metabolism-related proteins. These proteins include ANT (196), CrK (76, 197), glycerol kinase (198), HK (17, 20, 21, 24, 165, 189), C-Raf kinase (199), glyceraldehyde 3-phosphate dehydrogenase (41, 200), TSPO (201), and tubulin (202). Here, we concentrate on the interaction of VDAC1 with HK, which effectively couples OXPHOS and glycolysis, an important factor in cancer cell energy homeostasis (the Warburg effect).

#### Cancer Cell Bioenergetics and Apoptosis Are Regulated by VDAC1–HK Interaction

Cancer cells are well characterized by their high rate of glycolysis, designed to satisfy the heavy demands of transformed cells for metabolic intermediates (203). The mitochondrial-bound isoforms HK-I and -II use newly synthesized ATP to catalyze the phosphorylation of glucose to G-6-P. These enzymes are overexpressed in many cancers, including colon, prostate, lymphoma, glioma, gastric adenomas, and breast cancers (118, 132, 204–206). Both HK-II and HK-I bind to mitochondria (16, 17, 81, 133) and possess the hydrophobic N-terminal domain necessary for binding to mitochondria. The isoforms have quite different mechanisms of expression regulation (207).

Various studies, including site-directed mutagenesis, have demonstrated that VDAC1 is the mitochondrial-binding site of HK (17, 18, 21, 133, 145, 208). HK binding to VDAC1 allows direct coupling of mitochondrially generated ATP to incoming glucose, permitting mitochondria to synchronize the glycolytic flux with that of the TCA cycle and ATP synthase (1, 23, 165). In this way, the VDAC1–HK complex can regulate not only the glycolytic pathway but also other seminal metabolic pathways, such as the pentose phosphate shunt. The VDAC1–HK interaction was shown to be regulated by phosphorylation, possibly as a result of upregulation of glycogen synthase kinase 3β (GSK3β) (24) by protein kinase C, or in response to the cholesterol content of the OMM (81).

Hexokinase-I and HK-II were shown to function as anti-apoptotic proteins *via* binding to VDAC1, with their detachment enabling apoptosis activation (14, 17, 20, 21, 24, 81, 165, 189, 209, 210). Thus, the interaction of HK with VDAC1 points to HK function not only in cell metabolism but also as regulator of apoptosis. This dual role of HK makes the VDAC1–HK complex an attractive target for anti-cancer therapy (see below).

Hexokinase interaction with VDAC1 protects cells against apoptosis as activated by Bax or Bak (81, 119, 132, 209) and HK-I acts at the mitochondria to block TNF-induced apoptosis while conversely HK-I depletion accelerates the process (211). Importantly, it has been shown that single mutations in VDAC1 or when N-terminal truncated VDAC1 were expressed, HK-I showed no anti-apoptotic effect or reduction of channel conductance of bilayer-reconstituted (16, 17, 133, 210). In addition, mitochondria-bound HK-II inhibited Cyto *c* release and apoptosis as induced by Bax (209). Finally, VDAC1-based peptides, interacting with purified HK, were shown to prevent HK protection against apoptosis (20) (see VDAC1-Based Peptides As Potential Anti-Cancer Therapy).

#### HK Interaction with VDAC1 Offers Advantages to Cancer Cells

The advantages to cancer cells of HK binding to VDAC1 have been reviewed previously (12).

(a) *Production and access to energy and metabolites*: HK bound to VDAC1 has direct access to ATP newly produced in the mitochondria (212), facilitating the maintenance of a high glycolytic flux rate in tumors and, thus, increased energy and metabolite production (21). HK bound to VDAC1 is also less sensitive to inhibition by the product, G-6-P.
(b) *VDAC1-bound HK acts as an anti-apoptotic protein*: As presented above, HK-I and HK-II bound to VDAC1 function as anti-apoptotic proteins preventing Cyto *c* release and subsequent apoptosis (14, 17, 20, 21, 24, 81, 165, 189, 209, 210). HK also protects against Bax- or Bak-mediated apoptosis (81, 119, 209).
(c) *Regulation of ROS production/efflux from the mitochondria by HK*: ROS production is usually increased in cancer cells (213). HK, when associated with the mitochondria, reduced both mitochondrial ROS generation (214) and intracellular levels of ROS (105).
(d) *Increased synthesis and uptake of cholesterol*: HK binding to the mitochondria mediates increased synthesis and uptake of cholesterol into the mitochondria of cancer cells (81).

#### Disrupting VDAC1–HK Interaction As a Strategy to Interfere with Cancer Cell Growth and Induce Cell Death

The advantages for cancer cells presented above make the HK–VDAC1 complex an attractive target for anti-cancer therapy.

A number of agents, including 2-deoxyglucose (2-DG), 3-bromopyruvate (3-BP), an alkylating reagent, and lonidamine have been used to inhibit HK activity and disrupt glycolysis (215). In addition, the anti-fungal agents clotrimazole and bifonazole were shown to disrupt the HK-VDAC1 complex (189, 216).

However, glycolysis inhibitors can affect not only cancer cells but also normal tissues that use glucose as their main energy source (brain, retinae, and testis). Despite these concerns, targeting VDAC1–HK interaction proposed as a promising target for anti-cancer therapy (215, 217). Detachment of HK from VDAC1 impairs energy and metabolic homeostasis, including the coupling between glycolysis and mitochondrial metabolism, as well as enabling activation of apoptosis. Disturbing the VDAC1–HK interaction could, furthermore, influence cholesterol synthesis and distribution in the OMM (83).

Several agents have been identified that can dissociate the VDAC1–HK complex including HK-I- (218) and HK-II-derived peptides, clotrimazole (165, 216), a cell-permeable HK-II-based peptide (119), and MJ (189). Recently, synthetic VDAC1-based peptides (20, 145, 210) were shown to interact directly with HK-I and HK-II and lead to their dissociation from VDAC1 (see VDAC1-Based Peptides As Potential Anti-Cancer Therapy).

Since VDAC1-bound HK is essential for tumor cells, the detachment of HK from VDAC1 represents a novel therapeutic strategy to impair cancer metabolism and augment apoptosis (11, 12, 73, 215).

### Interaction of VDAC1 with Bcl-2 Family Members

The resistance of cancer cells to apoptosis involves a variety of strategies. One of these involves the overexpression of anti-apoptotic proteins of the Bcl-2 family, which contributes to disease progression and drug resistance (129, 219–221) The Bcl-2 family comprises pro-apoptotic (e.g., Bid, Bax, Bim, and Bak) and anti-apoptotic (e.g., Bcl-2 and Bcl-x_L_, Mcl-1) members that up- or downregulate apoptosis, respectively (222). Mcl-1 has been shown to interact directly with VDAC1 to increase mitochondrial Ca^2+^ uptake and ROS generation (223).

The mechanisms by which Bcl-2 family proteins regulate apoptosis involve interactions with mitochondria and control of OMM permeability. Regulation of apoptosis by interactions of VDAC1 with these proteins has been reported in a number of studies (16, 19, 20, 22, 139, 224, 225). VDAC1 has been shown to interact with Bax/Bak (142, 226), Bcl-2 (16, 19, 51), Bcl-xL (18, 19, 22, 25, 48, 96, 224, 225, 227), and with Bax and Bim (26, 158, 225), such that anti-VDAC antibodies inhibited Bax- and Bim-induced release of Cyto *c* (136). In addition, Bcl-2 and Bcl-xL block As_2_O_3_-induced VDAC1 dimerization (137). BH4 oligopeptides derived from Bcl-2 and Bcl-xL were able to inhibit VDAC1 activity in liposomes, even in the presence of a pro-apoptotic protein, such as Bax (224). Bcl-2 and Bcl-xL were shown to reduce the conductance of native but not mutated VDAC1, as well as to protect cells expressing native but not mutated VDAC1 against apoptosis (18, 19). In addition, activation of Bax by cisplatin was prevented in cells silenced for VDAC1 expression (25, 139). Finally, Bid was shown to interact with VDAC1 as reflected in the decrease in VDAC1 conductance (228).

Thus, VDAC1 binds members of the Bcl-2 family proteins thereby regulating their effects on apoptosis. Hence, interfering with such interactions could facilitate apoptosis induction and enhance the therapeutic effect of chemotherapeutic agents.

### Interaction of VDAC1 with Other Proteins

Translocator protein is closely associated with VDAC1 (229) and this association allows ROS generated *via* TSPO to affect VDAC1 (201, 230). In addition, overexpression of TSPO inhibits VDAC1 expression, while VDAC1 expression level was increased upon silencing of TSPO in endothelial cells (231).

A number of cytoskeletal proteins have been reported to interact with and regulate VDAC1. These proteins include the following.

*Gelsolin* (Gsn) is a Ca^2+^-dependent protein that regulates actin assembly and disassembly. Human (h)Gsn has pro-apoptotic or anti-apoptotic activity, depending on the cell type (232). hGsn inhibited VDAC1 channel activity and interacted with VDAC1-containing liposomes in a Ca^2+^-dependent manner to inhibit Cyto *c* (232).

*Tubulin*, co-immunoprecipitated with VDAC1 (233) and the association was also demonstrated by tubulin-induced VDAC1 closure (202), an effect thought to sustain the Warburg effect (234). It is proposed that tubulin, VDAC1, and MtCK form a super-complex that is structurally and functionally coupled to the ATP synthasome (235).

*Microtubule-associated protein 2* was shown by affinity chromatography to bind VDAC1 (236).

The mitochondrial anti-viral signaling protein, also known as IPS-1, VISA, or Cardif (237) and localized in the OMM, was demonstrated to modulate VDAC1 protein stability *via* the ubiquitin–proteasome pathway (238).

*Superoxide dismutase 1 (SOD1)* mutated protein, which is associated with ALS, and reduced VDAC1 channel conductance (239) and altered the interaction between VDAC1 and Bcl-2 (240).

*Endothelial NO synthase (eNOS)* was also found to bind VDAC1, with this amplifying eNOS activity intracellularly in a Ca^2+^-dependent manner (241).

Several additional proteins were shown or proposed to interact directly with VDAC1. These include PBP74 (heat-shock protein peptide-binding protein 74), also known as mtHSP70/GRP75/mortalin (242), and GRP78, a 78-kDa glucose-regulated protein that forms a complex with vaspin. The complex of GRP78, and VDAC on the plasma membrane, promotes proliferation, inhibits apoptosis, and protects against vascular injury in diabetes mellitus (243). Other proteins that can interact with VDAC are the ORDIC channel, actin (244), Nek1 (NIMA-related protein kinase 1) (173), aldolase (41), Tctex-1/DYNLT1 (dynein light chain) (242), CRYAB (α-crystallin B) (245), and α-synuclein (246).

The pro-apoptotic protein BNIP3 was shown to interact with VDAC1 to induce mitochondrial release of endonuclease G (247) and VDAC1 co-immunoprecipitated with the L-type Ca^2+^ channel (248).

A total of 44 VDAC1 interacting genes were identified as being commonly differentially expressed between normal and tumor tissues in human carcinomas (249).

In summary, VDAC1 serves as a central hub for responses to cellular signaling and the effects may be mediated by interaction with many proteins (Figure 1), indicating the central role played in cell metabolism and apoptosis.

## Alterations in VDAC1 Expression Level in Cancer

Voltage-dependent anion channel 1 is highly expressed in many cancer types compared to the levels in normal cells. VDAC1 overexpressed in several cancer cell lines relative to fibroblast cell line (250) and, in ascites hepatoma AH130 cells, the three VDAC isoforms expression levels were significantly higher than in normal liver cells. Higher VDAC1 levels were connected to primary malignancies of the biliary tract (251), and were found in gastric cancer cells (252). Both VDAC1 mRNA and protein levels were upregulated in H358 cells (253). Induction of Cyto *c* release by G3139 in several melanoma and prostate cancer cell lines was found to be correlated with VDAC1 expression levels (254). In myeloma cells, CD45 expression was accompanied by elevated VDAC1 expression that sensitized the cells to a diverse set of apoptotic stimuli (255). These differences are not surprising in light of the of VDAC1 functions in cell metabolism and energy production, systems particularly important for cancer cells proliferation. Overexpressed VDAC1 presents anchoring sites for the cancer overexpressed HK and for Bcl-2 and Bcl-xL, interactions that are important for their anti-apoptotic activities (see Modulation of VDAC1-Mediated Apoptosis and Metabolism *VIA* Interacting Proteins).

Overexpression of VDAC1 was detected in tissue arrays for thyroid, lung, cervix, ovary, pancreas, melanoma, and glioblastoma cancers as well as in lung tissue samples taken from healthy and tumor-containing areas of the same patient (12). Similar results were obtained in other studies of breast, colon, liver, lung, pancreatic, and thyroid cancers (249) and lung tumors (256). VDAC1 was also found to be overexpressed in peripheral blood mononuclear cells (PBMCs) from chronic lymphocytic leukemia (CLL) patients, as compared to PBMCs from healthy donors (257). Cervical cancer patients with high VDAC1 displayed higher rates of recurrence and poorer overall survival than those with low VDAC1 (258). VDAC also appears to be a potential marker for the diagnosis of colorectal cancer (259) and gastric cancer (260). VDAC1 overexpression was demonstrated in lung cancer, where C-terminally truncated VDAC1 (VDAC1-ΔC) was present in tumor cells exposed to hypoxia in 50% of 46 patients with lung cancer (261). In addition, a significant positive correlation exists between the levels of VDAC1 and the histological grade of breast cancer (262) as well as poor prognosis of primary lung adenocarcinoma (263) and of non-small cell lung cancer (NSCLC) patients (264). In NSCLC VDAC1 was proposed as a potential predictor of poor outcome in the diseases early stage (264). In addition, tumor progression and sensitivity to chemotherapy was correlated with VDAC1 expression (263, 265). Thus, VDAC1 expression levels can serve as a biomarker for cancer development, treatment efficacy, and as a predictor of poor outcome.

## Unraveling VDAC1-Based Therapies

As already discussed, VDAC1 offers a unique target for anti-cancer therapies because of its role as a key regulator of energy and metabolism and apoptosis. VDAC1-based therapeutic strategies include RNA interference (RNAi) designed to downregulate VDAC1 expression levels and cause growth arrest, as well as VDAC1-based peptides that impair energy homeostasis and minimize the self-defense mechanisms of cancer cells and small molecules that induce apoptosis. Together, such anti-cancer therapies are expected to be highly effective, even in drug-resistant tumors.

### Silencing VDAC1 Expression by Short Hairpin RNA (shRNA) or siRNA As a Tool to Reprogram Cancer Cell Metabolism

The role of VDAC1 as a key regulator of the cellular metabolic and energy reprogramming processes essential to cancer survival (1, 11, 12, 61) makes targeting VDAC1 an attractive strategy for anti-cancer therapy. As one approach, overexpressed VDAC1 can be downregulated by using RNAi, including shRNA and siRNA.

#### VDAC1 Silencing Using shRNA and siRNA As a Strategy for Cancer Therapy

We have demonstrated that downregulation of VDAC1 expression by hVDAC1-shRNA disrupts energy production, arrests cell growth, and inhibits tumor development in an animal model, illustrating the essential role of VDAC1 in energy production and cell growth (53, 266). Furthermore, tumors developed into nude mice from HeLa cervical cancer cells stably expressing shRNA directed against hVDAC1, strongly inhibited the development of tumors (266). Indeed, silencing VDAC1 by shRNA blocked TRAIL-induced mitochondrial apoptosis, suggesting that expression of VDAC1 is required for caspase-8 activation (267). The use of hVDAC1-shRNA permitted the demonstration of VDAC1 involvement in arsenic trioxide-, ascorbic acid-, and disulfiram (AAA)-induced aponecrosis and the switch from apoptosis to the aponecrosis death pathways (268). The shRNA also reduced cell proliferation and migration of cervical cancer cells, and increased ROS production (258). Stable expression of hVDAC1-shRNA stimulated NLRP inflammasome activators and augmented caspase-1 and IL-1β secretion in THP cells (269). These multiple effects of VDAC1 silencing point to an additional role for VDAC1 as a central protein in regulating cell signaling.

Silencing VDAC1 expression by a single siRNA specific to the human VDAC1 (si-hVDAC1) sequence resulted in cell proliferation and cancer cell growth inhibition both in cell cultures and *in vivo* animal models (75, 270). In addition, siRNA at nanomolar concentrations silenced VDAC1 expression in many tested cell lines, inhibited cell growth (over 90%) and decreased ATP levels (53, 75, 266). In *in vivo* experiments using a xenograft lung cancer mouse model, si-hVDAC1 inhibited tumor growth and even caused tumor regression (75).

Recently (270), we demonstrated that depleting VDAC1 by si-hVDAC1 assaults critical functional nodes in the oncogenic network of GBM tumors, leading to a multi-pronged attack on cancer hallmarks, reversing cancer-reprogrammed metabolism, thereby inhibiting cell proliferation, tumor growth, EMT, and angiogenesis, and also targets GSCs, leading to their differentiation into neuronal-like cells.

Other studies from our group (271) using a GBM xenograft mouse model showed that upon VDAC1 depletion, several pro-apoptotic proteins were overexpressed yet apoptosis was not induced. This suggests that essential players of the apoptosis executioner pathways may serve dual functions, acting either to kill cells or to promote differentiation, depending on the energy level of the cell.

Another connection between VDAC1 and cell energy signaling was demonstrated in recent studies showing that VDAC1 is a direct target of the anti-fungal agent, itraconazole, and that VDAC1 is a key mediator of the inhibition of mTOR and endothelial cell proliferation by the AMPK signaling pathway (270, 272).

si-hVDAC also markedly decreased HIF1-α levels and tumor growth in U-87MG and U-118MG cancer cells (270). These findings suggest a relationship between HIF1-α and VDAC1 expression and tumor growth.

#### MicroRNA (miRNA) Acts *via* Modification of VDAC Expression Levels

MicroRNAs belong to a class of small, non-coding, regulatory RNAs which bind to the 3-UTR of target mRNAs to reduce target protein levels.

The level of miR-7 has been shown to be downregulated in various cancer cells, such as GBM (273), breast cancer (274), urothelial carcinoma (275), gastric tumors (276), pancreatic cancer (277), colorectal cancer (278), and in hepatocellular carcinoma tissues, as compared to adjacent non-tumor tissue (279). These findings suggest that miR-7 has a tumor suppressor function. Several studies have demonstrated that the miR-7 regulates the function of the mitochondrial PTP by downregulating VDAC1 expression. Overexpressing VDAC1 without the 3-UTR significantly abolished the protective effects of miR-7 against 1-methyl-4-phenylpyridinium ion (MPP)-induced cytotoxicity and mitochondrial dysfunction (280).

Proteomic profiling of cells showed that overexpression of miR-29a also resulted in downregulation of VDAC1 and the VDAC2 protein (281).

Thus, considering the high expression level of VDAC1 in tumors and the specificity of si-hVDAC1 in inhibiting cancer cell and tumor growth, silencing VDAC1 expression can be considered as a novel strategic therapeutic approach to treat cancer.

### VDAC1-Based Peptides As Potential Anti-Cancer Therapy

#### VDAC1-Based Peptides—Development and Cell Death Induction

Cancer cells share several features that distinguish them from normal cells, including avoiding apoptosis, thereby drug resistance (282). Indeed, defects in the regulation or even evasion of apoptosis are hallmarks of cancer (1, 11, 61, 115). To avoid apoptosis, cancer cells developed several strategies, such as overexpression of anti-apoptotic proteins, such as the Bcl-2 family of proteins and HK, to prevent the release of Cyto *c* from mitochondria (1, 11, 124).

To mediate their anti-apoptotic activities, HK, Bcl-2, and Bcl-xL interact with VDAC1 (16–20, 133). Therefore, we engineered VDAC1-based peptides designed to interfere with these interactions. We identified those VDAC1 domains and amino acid residues important for the interaction of VDAC1 with HK, Bcl-2, and Bcl-xL and designed VDAC1-based peptides specifically targeting these interactions (15–20, 22, 133, 224, 225). These peptides are designed to serve as “decoy” peptides that compete with VDAC1 for the HK-, Bcl-2-, and Bcl-xL–VDAC1 interactions and consequently abolish their anti-apoptotic activities. As the VDAC1-based peptide target intracellular proteins, several cell-penetrating peptides were developed.

**Figure 5:**
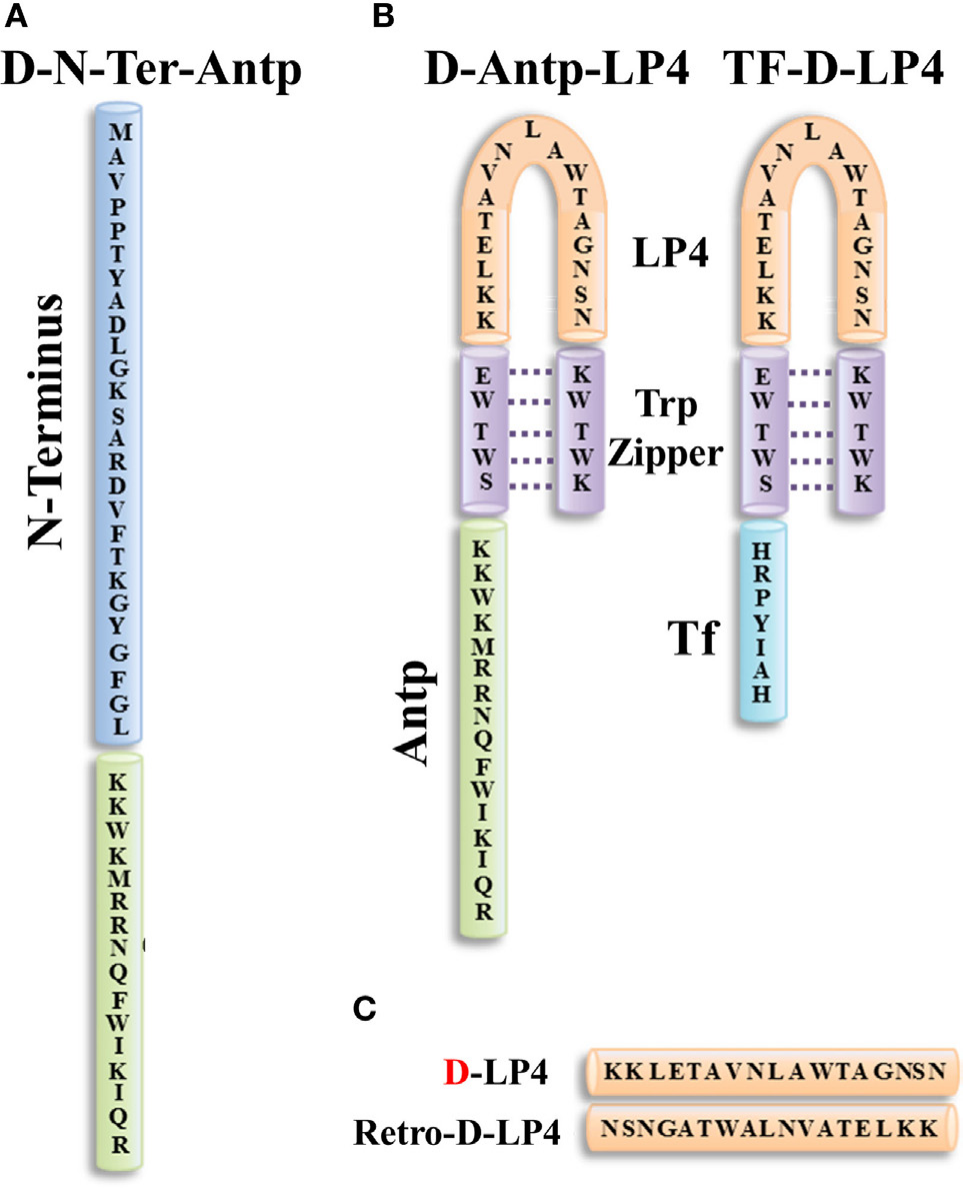
Structure of VDAC1-based peptides. Schematic illustration of D-N-terminus-Antp (D-N-Ter-Antp) **(A)** and D-Antp-LP4 and Tf-D-LP4 **(B)** peptides. The VDAC1-derived sequences N-terminus and LP4 are in blue and orange, respectively. The cell-penetrating peptide Antp in green and Tf is in light blue. The loop shape of LP4 stabilized by a tryptophan zipper (Trp) is in purple. The N-terminus of LP4 and Antp is composed of d-amino acids. **(C)** The sequences of D-LP4 and retro-D-LP4.

VDAC1 derived sequences corresponding to the N-terminal domain (N-Ter) and a VDAC1 sequence partially exposed to the cytosol (LP4) were fused to Antp (penetrating), a 16 residue-long sequence from the *Drosophila* antennapedia-homeodomain, to yield the Antp-LP4 and N-Ter-Antp peptides (Figure 5). These peptides promoted cell death in a variety of genetically characterized cell lines derived from different human cancers (70, 283).

*Antp-LP4*—this is a loop-shaped cell-penetrating peptide comprising the SWTWE sequence at the N-terminal end and the KWTWK sequence at the C-terminal end of a VDAC1-derived sequence (LP4, residues 199–216). This generates a tryptophan zipper and a stable β-hairpin conformation (284), mimicking the LP4 loop in the native VDAC1 protein, which is fused to Antp. Antp-LP4 peptide prevented the anti-apoptotic effects of HK, Bcl-2, or Bcl-xL (16–20, 133) and induced cell death in several cancer cell lines, while being less effective in non-cancerous cells (70).

*N-Ter-Antp*—in this peptide, the N-terminal sequence was selected based on the findings that N-terminal domain-truncated VDAC1 had lost the ability to bind HK, Bcl-2, or Bcl-xL (16–20, 133). N-Ter-Antp peptide interacted with HK, Bcl-2, and Bcl-xL and inhibited their anti-apoptotic effects (16–20, 133).

*Tf-D-LP4 peptide*—this peptide comprises a VDAC1-derived sequence (LP4), with the tryptophan zipper fused to a cell penetrating peptide, a human transferrin receptor (hTfR) (CD71)-recognition sequence, HAIYPRH (Tf) (Figure 5). hTfR is overexpressed (up to 100-fold) in liver, pancreatic, prostate, GBM, and other cancers (285), relative to their normal counterparts and is also highly expressed in the BBB (286).

Based on results obtained using over 40 versions of cell-penetrating peptides, several modifications were introduced into the VDAC1-based peptides to in order to address peptide efficacy, stability, cell penetration, and specificity toward cancer cells as described below.

The effects of the selected designed VDAC1-based peptides were tested on 42 different cancer cell lines and found to induce cell death regardless of cancer type or mutation status, with specificity toward cancerous cells (16, 17, 19, 20, 70, 133). VDAC1-based peptides (N-Ter and LP4 derived sequences) were shown to limit Ca^2+^ uptake into the mitochondrial matrix and inhibit ROS generation in lung cancer cells (223). In addition, the use of VDAC1-based peptides (residues 10–30) suggests that VDAC1 is part of a system controlling cell proliferation in neuroblastoma cells *via* its interaction with glucose-regulated protein 78 (GRP78) (287).

#### The Mode of Action of VDAC1-Based Peptides

**Figure 6:**
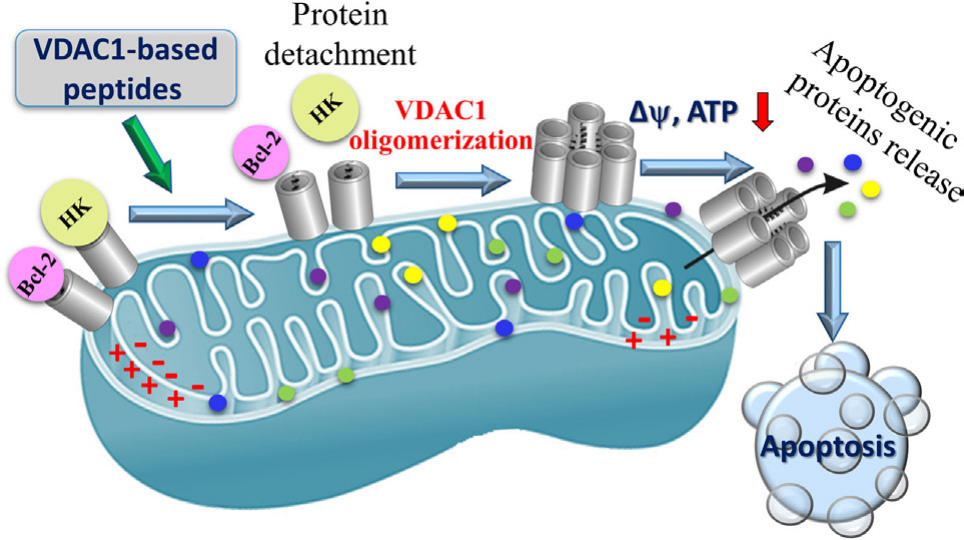
Proposed mode of action of VDAC1-based peptides leading to mitochondria-mediated cell death. VDAC1 is overexpressed in the mitochondria of cancer cells and associated with hexokinase (HK) and Bcl-2, Δψ is maintained, the cell remains in homeostasis with respect to energy production and is protected from apoptosis. VDAC1-based peptides interact with anti-apoptotic proteins HK and Bcl-2 causing the proteins to disassociate from VDAC1, and leading to Δψ dissipation, decreased ATP production, mitochondrial dysfunction, VDAC1 oligomerization, and Cyto *c* release. These events ultimately lead to cell death.

Our results suggest that the VDAC1-based peptides lead to: (i) impaired cell metabolism and energy homeostasis; (ii) negation of the anti-apoptotic activities of HK, Bcl-2, and Bcl-xL, and (iii) induction of massive apoptosis (16, 17, 70) (Figure 6).

Voltage-dependent anion channel 1-based peptides were found to impair cell metabolism and energy homeostasis. Altered energy metabolism, including the use of glucose *via* glycolysis as an energy source, is a common feature of most malignant tumors (115, 288). Mitochondrial-bound HK is highly expressed in glycolytic cancer cells (289), supporting aerobic glycolysis (23) but also conferring stability to mitochondria (290) and resistance to apoptosis (14, 17, 20, 21, 24, 81, 165, 189, 209, 210). As already discussed, VDAC1 is overexpressed in many cancer types (see Alterations in VDAC1 Expression Level in Cancer) (12) and, thus, presents anchoring sites for overexpressed HK, allowing direct access to mitochondrial ATP and an increased glycolytic rate (1). The VDAC1-based peptides interact with and detach HK from its binding site in VDAC1, leading to decreased glycolysis, and decreases in ΔΨ and cellular ATP levels (16, 20). Thus, detachment of mitochondria-bound HK leads to a disruption of the cellular energetics status of cancer cells.

VDAC1-based peptides were found to prevent the anti-apoptotic activity of anti-apoptotic proteins to induce apoptosis. As already described above, cancer cells utilize a variety of strategies to limit or avoid apoptosis, including overexpression of anti-apoptotic proteins, such as members of the Bcl-2 family of proteins and HK. VDAC1 binds HK, Bcl-2, and Bcl-xL (16, 18–20, 51, 96, 142, 224, 227). The VDAC1-based cell-penetrating peptides N-Ter and LP4 were shown to interact directly with purified anti-apoptotic proteins and, after penetrating into cells, antagonized their anti-apoptotic activities (18–20). These findings suggest that VDAC1-based peptides interfere with the binding of anti-apoptotic proteins to VDAC1 thereby permitting apoptosis induction.

VDAC1-based peptides induced VDAC1 oligomerization, release of Cyto *c* and other molecular hallmarks of apoptosis, promoting membrane blebbing, phosphatidylserine surface exposure, and nuclear condensation and fragmentation (20, 70) (Figure 6). This multiple mode of peptide activities may explain their high potency and specificity toward tumor cells.

#### The Effects of VDAC1-Based Peptides on Cancer—*Ex Vivo* and *In Vivo* Studies

Peptide activity was tested in an *ex vivo* study using samples from CLL patients (70). CLL is characterized by a clonal accumulation of mature neoplastic B cells that are resistant to apoptosis (291). VDAC1-based peptides (Antp-LP4 and N-terminal-Antp) selectively killed PBMCs obtained from CLL patients but not from healthy donors (70). The ability of the peptides to induce cell death was well correlated with the amount of the cancer cells. The mode of action of the peptides on CLL involves inhibition of energy production and apoptosis induction.

In sub-cutaneous and intracranial xenograft mouse models of GBM, the VDAC1-based peptides, N-Ter and Tf-D-LP4, were found to disrupt cell metabolism and energy homeostasis to inhibit tumor growth, invasion, stemness, and induce apoptosis (71). Peptide-treated tumors downregulated metabolism-related enzymes and transporters, and elevated the levels of apoptotic proteins, such as p53, Cyto *c*, and caspases. In agreement with the results obtained with cells in culture, the peptides acts by impairing energy and metabolism, interfering with the actions of anti-apoptotic proteins, and inducing cell death (71). VDAC1-based peptides, thus, offer an affordable and innovative new conceptual therapeutic paradigm that can potentially overcome chemoresistant, invasive, GBM cancer stem cells, and reduce relapse rates.

To summarize, VDAC1-based peptides act relatively fast and at low concentrations to induce cell death in a variety of cancer cell lines, irrespective of the origin of the cancer or carried mutations. This is very important in view of tumor heterogeneity, metastatic transformation, and modifications acquired during tumor development.

## VDAC Involvement in Diseases Other Than Cancer

### Neurodegenerative Diseases, Mitochondria, Apoptosis, and VDAC

Impaired mitochondrial function has been reported for most neurodegenerative diseases, such as Parkinson’s disease, Huntington’s disease, ALS, and AD (292). Recent studies have shown that such disorders share characteristics of mitochondria-mediated apoptotic death (293).

Mitochondrial dysfunction is an early event in AD pathogenesis, as reflected by reduced metabolism, disruption of Ca^2+^-homeostasis, increased free radical production, and lipid peroxidation (294–296). Aβ also affects mitochondrial respiration (297) and activates Cyto *c* release, resulting in apoptosis (298). Importantly, Aβ does not cause toxicity in cells depleted of mitochondria (299). High-levels of VDAC1 were demonstrated in the dystrophic neurites of Aβ deposits in AD post-mortem brains and amyloid precursor protein transgenic mice (300). VDAC1 was shown to participate in Aβ-induced toxicity (45, 301, 302) where Aβ–VDAC1 interactions are toxic to AD-affected neurons (303) and VDAC1 interactions with Aβ and phosphorylated Tau lead to mitochondrial dysfunction (302). Recently (45), we demonstrated that Aβ interacts directly with VDAC1, specifically with the N-terminal region. Moreover, VDAC1 is required for Aβ entry into the cell, as well as Aβ-mediated apoptosis, since Aβ cell penetration and toxicity were prevented in cells depleted of VDAC1 using siRNA. Finally, an increase in nitrated VDAC1 in AD was reported, reflecting oxidative damage to VDAC (304), and possibly affecting cell energy and metabolite homeostasis (305). The involvement of plasmalemmal VDAC in AD was also proposed (301, 306).

These findings point to VDAC1 as a potential target for novel therapeutic strategies for AD.

### Cardiovascular Diseases, Mitochondria, Apoptosis, and VDAC

The loss of cardiac myocytes plays a critical role in the pathogenesis of cardiovascular disorders. Activation of the mitochondrial pathway of apoptotic cell death has been implicated in ischemia/reperfusion injury involving the release of Cyto *c* from mitochondria, followed by activation of caspase-9 in the myocardium (307). VDAC1 levels were increased in cardiomyoblast H9c2 cells that were differentiated in the presence of all-trans retinoic acid (308). The increased susceptibility of differentiated cells to mitochondrial-mediated cell death may be related to the increase in VDAC1 levels.

### Type 2 Diabetes (T2D) and VDAC1

In T2D, beta cell decompensation develops when insulin secretion fails to balance insulin resistance. Hyperglycemia was found to increase VDAC1 expression in pancreatic β-cells (309) and in the kidney (310). VDAC1 levels were also increased in mouse coronary vascular endothelial cells isolated from diabetic mice. This was associated with increased mitochondrial Ca^2+^ concentration, mitochondrial O_2_^−^ production, and PTP opening activity (311). Finally, since glucose-stimulated insulin secretion depends on the generation of ATP and other metabolites in the mitochondria (312) and since VDAC1 regulates energy and metabolism, it follows that VDAC1 is required for insulin secretion.

## Perspectives

In this review, we highlighted the cancer–mitochondria–metabolism–apoptosis-VDAC1 axis. As Otto Warburg noted almost a century ago, cancer cells frequently share bioenergetics abnormalities, regardless of their cellular or tissue origin. Furthermore, the view of cancer as primarily a metabolic disease can impact any approach to cancer management and prevention. As a mitochondrial gatekeeper and overexpressed in cancer VDAC1 is a very attractive emerging anti-cancer drug target.

Another hallmark of cancer cells is their ability to avoid apoptosis by activating anti-apoptotic mechanisms associated with drug resistance, including overexpression of anti-apoptotic proteins that interacts with VDAC1. As such, interfering with this association would allow for activation of the mitochondrial pathway of apoptosis and allow for apoptosis induction by anti-cancer drugs.

Most importantly, VDAC1, in the OMM, serves as a hub protein, with its interactome, including over 150 proteins involved in metabolism, apoptosis, signal transduction, and anti-oxidation, as well as DNA- and RNA-associated proteins, that together mediate and/or regulate metabolic, apoptotic, and other processes in normal and diseased cells. Thus, VDAC1 can be considered as a key protein not only in metabolism and apoptosis regulation but also as a link between the energy, redox, and signaling pathways in mitochondria and other cell compartments. Thus, targeting these VDAC1–protein interactions may interfere with a variety of processes, such as cellular energy homeostasis, apoptosis, and other activities and signaling pathways in cancer.

Finally, VDAC1 silencing in tumors leads to reprogramed metabolism and results in alterations in the transcription factors that regulate signaling pathways associated with cancer hallmarks affecting angiogenesis, EMT, invasiveness, stemness, and induced differentiation. This suggests that VDAC1 plays a key role in cancer cell fate by controlling the cross-talk between metabolism and oncogenic signaling networks, most likely affecting the interplay between metabolism and epigenetics. VDAC1 overexpression is, moreover, a signature of most cancers and can serve as a prognostic biomarker to predict clinical outcome in diversified human cancers. Thus, a comprehensive understanding of the exact molecular basis of the complex signaling networks activated by VDAC1 over- or downregulation in the tumor that forms a pro- or anti-tumorigenic milieu is required.

## Author Contributions

All authors contributed to this review by writing certain sections of the review and preparing the illustrations. All the authors read and approved the final manuscript and agreed to be accountable for all aspects of the work.

## Conflict of Interest Statement

The authors declare that the research was conducted in the absence of any commercial or financial relationships that could be construed as a potential conflict of interest.

## Abbreviations

AIF, apoptosis-inducing factor; ANT, adenine nucleotide translocase; APAF-1, apoptosis protease-activating factor 1; Bcl-2, B-cell lymphoma 2; caspase, cysteinyl/aspartate-specific protease; CLL, chronic lymphocytic leukemia; CyP D, cyclophilin D; Cyto *c*, cytochrome *c*; DIDS, 4,4′-diisothiocyanostilbene-2,2′-disulfonic acid; ER, endoplasmic reticulum; FNQs, furanonaphthoquinones; G-6-P, glucose-6-phosphate; HK, hexokinase; IMM, inner mitochondrial membrane; IP_3_Rs, inositol-1,4,5-trisphosphate receptors; LDH, lactate dehydrogenase; MAC, mitochondrial apoptosis-inducing channel; NADH, reduced nicotinamide adenine dinucleotide; NSCLC, non-small cell lung cancer; OMM, outer mitochondrial membrane; PDH, pyruvate dehydrogenase; PLB, planar lipid bilayer; PT, permeability transition; PTP, permeability transition pore; RNAi, RNA interference, shRNA, short-hairpin RNA; ROS, reactive oxygen species; RuR, ruthenium red; SR, sarcoplasmic reticulum; STS, staurosporine; TNF-α, tumor necrosis factor alpha; TSPO, translocator protein; VDAC, voltage-dependent anion channel.
